# Beyond the cut: a cross-sectional analysis of the long-term clinical and functional impact of cesarean section scars

**DOI:** 10.61622/rbgo/2025rbgo55

**Published:** 2025-07-15

**Authors:** Laila Lídia Faria Almeida, Gabriel Lage Neves, Matheus Eduardo Soares Pinhati, Rivia Mara Lamaita, Eduardo Batista Cândido, Agnaldo Lopes da Silva

**Affiliations:** 1 Universidade Federal de Minas Gerais Faculdade de Medicina Belo Horizonte MG Brazil Faculdade de Medicina, Universidade Federal de Minas Gerais, Belo Horizonte, MG, Brazil.; 2 Faculdade Ciências Médicas de Minas Gerais Belo Horizonte MG Brazil Faculdade Ciências Médicas de Minas Gerais, Belo Horizonte, MG, Brazil.; 3 Universidade Federal de Minas Gerais Departamento de Ginecologia e Obstetrícia Belo Horizonte MG Brazil Departamento de Ginecologia e Obstetrícia, Universidade Federal de Minas Gerais, Belo Horizonte, MG, Brazil.

**Keywords:** Cesarean section, Postpartum period, Scars, Cicatrix, Quality of life, Multidisciplinary care team, Self care, Personal satisfaction, Treatment outcome, Surveys and questionnaires

## Abstract

**Objective:**

This study aims to evaluate the clinical and functional implications of cesarean section (CS) skin scars on women's lives, emphasizing the relationship between scar characteristics and quality of life.

**Methods:**

In this cross-sectional study, women older than 18 years old who had undergone CS with a Pfannenstiel incision within the past 6-36 months were evaluated. The Patient Scar Assessment Questionnaire (PSAQ) and the Patient and Observer Scar Assessment Scale (POSAS) were used to assess clinical scar parameters. Functionality was appraised using eight questions derived from the International Classification of Functionality, Disability, and Health (ICF). The association between scar appearance scores and functionality questions was analyzed statistically.

**Results:**

Ninety-six women were assessed, revealing that appearance and satisfaction with appearance had the worst scores on the PSAQ. Scar irregularity was the most frequently reported scar characteristic on the POSAS. On the ICF-derived questionnaire, the "self-care" domain was the most affected, with women reporting problems on activities such as choosing swimwear and lingeries. There was a significant correlation (p < 0,05) between the women dissatisfaction with the scar appearance and impairment in ‘interpersonal interactions and relationships’ and ‘self-care’, as the "domestic life" domain wasn't affected.

**Conclusions:**

Cesarean section skin scars can lead to dissatisfaction and functional impairments, affecting women's quality of life. These findings underscore the necessity for multidisciplinary care and thorough discussions about delivery methods to improve post-surgical outcomes.

## Introduction

The cesarean section (CS) is the most prevalent obstetric surgical intervention, and it serves as a critical life-preserving procedure when medically warranted. However, its rise in frequency has brought to light a spectrum of associated risks and complications with both short- and long-term implications.^(1)^

One of the most important complications related to CS is the increase in morbidity and mortality rates when compared to vaginal deliveries.^(2)^ Moreover, the risks extend to increased probabilities of infection, uterine rupture, abnormal placentation, ectopic pregnancy, stillbirth, and preterm birth. These clinical risks, although significant, represent only a fraction of the potential adverse outcomes women may face.^(3,4)^

Notably, postoperative scarring is an anticipated outcome. However, the clinical and functional consequences of such scars have not been properly explored, as the implications of CS scars transcend mere physical remnants, as they are complex and multifaceted and involve physical and social dimensions.^(1-4)^

In a society where women's physical appearance is highly scrutinized, living with a CS scar can be particularly challenging. Scars can be not only painful but also aesthetically distressing and disfiguring and can impair psychosocial and functional capabilities.^(5)^ Negative aesthetic perceptions, such as hyperpigmentation and hypertrophy, may lead to dissatisfaction and embarrassment, critically impacting daily activities and social participation.^(6)^

The functional repercussions of CS scars are an additional concern, potentially interfering with the quality of life.^(7)^ The World Health Organization's International Classification of Functionality, Disability, and Health (ICF) provides a framework for understanding functionality in terms of body structures and functions, activities, and participation in social contexts.^(8)^ While medical complications of CS scarring are well-documented, the broader effects on women's social and sexual lives remain insufficiently addressed.^(3)^

A multidisciplinary approach to the care of women undergoing CS is imperative, particularly during the post-surgical period when concerns regarding scar healing may emerge. The need to focus on the clinical and functional effects of CS skin scars represents a neglected aspect of postoperative complications, which may significantly impact women's quality of life, social integration, and sexual function. This study, therefore, aims to fill this gap by evaluating the clinical and functional long-term impacts of CS skin scars on women.^(3)^

## Methods

A cross-sectional study, guided by the STROBE statement and checklist, was conducted in an university in Belo Horizonte, Brazil.^(9)^ Recruitment of participants and data collection occurred between March 2017 and October 2019. The patients were recruited through social media platforms and the data was subsequently collected through phone calls by a trained interviewer.

This study included women older than 18 years old who had undergone CS with a Pfannenstiel incision within the past 6-36 months. Women who were pregnant, who had other prior abdominal surgeries, who had any history of aesthetic or surgical scar treatment or who had received radiation therapy post-CS were not considered eligible to participate.

For data collection, a form of demographic, clinical and surgical characteristics, created by the reseachers, was applied at first. It contemplates information about marital status, ethnicity, educational level, body mass index (BMI), smoking, presence of chronic diseases, interest in aesthetic scar treatment, number of months since the CS, scar length, number of previous CS, timing of surgery (non-elective or elective) and health system (private or public).

Then, two questionnaires were used to analyse the subjective impact of CS scars: the Patient Scar Assessment Questionnaire (PSAQ) and the Patient and Observer Scar Assessment Scale (POSAS). The PSAQ only gauges the impact of CS scars from the patient´s perspective. It consists of 28 items across four subscales: appearance, symptoms, satisfaction with appearance and satisfaction with symptoms. Each item of the PSAQ has a score that ranges from 1 (best score) to 4 (worst score).^(10)^ The POSAS gauges the impact of CS scars from both the patient´s and the observer´s perspective and includes two subscales: the Observer Scar Assessment Scale (OSAS) and the Patient Scar Assessment Scale (PSAS). The OSAS consists of 6 items (vascularity, pigmentation, thickness, relief, pliability and surface area) with scores that range from 1 (normal skin) to 10 (worst scar imaginable). The PSAS also consists of 6 itens (pain, itching, pigmentation, thickness, relief, pliability and surface area) with scores that also range from 1 to 10.^(11)^ The final results of the PSAQ and the POSAS subscales are expressed through their total scores, which consist of the sum of the scores of all their items. Both questionnaires have been previously validated for the Brazilian cultural context and are available in portuguese.^(12,13)^

Finally, functionality was evaluated using an eight-item questionnaire derived from the WHO's International Classification of Functionality, Disability, and Health (ICF). This questionnaire gauges the scar´s impact on three domains: "interpersonal interactions and relationships" (3 items assessing the scar´s impact on being naked in front of your partner, on sex life and on dating), "self-care" (4 items assessing the scar´s impact on shaving and wearing clothes, bikinis and lingeries) and "domestic life" (a single item assessing the scar´s impact on performance of domestic actitivities). The responses of the ICF-derived questionnaires range from ‘no problem’ to ‘extreme problem’ and reflect the degree of the scar´s impact on those daily life activities.^(8)^

We referenced the DATASUS (The IT Department of the Unified Health System of Brazil) database to ascertain the number of women who had undergone CS during the specified period, determining a minimum sample size of 96 to ensure a robust analysis with a 10% margin of error and 95% confidence level.

Data analysis was performed using R software. The association between qualitative variables was examined using the chi-square test. The Kruskal-Wallis test, a non-parametric alternative to the ANOVA, was utilized to analyze associations between mean scores, with an alpha error of 5% denoting statistical significance (version 2.5019’ Team 2019. RStudio: Integrated Development for R. RStudio, Inc., MA UR).

This study was approved by the Ethics and Research Committee of the Federal University of Minas Gerais (COEP/UFMG) (*Certificado de Apresentação de Apreciação Étic*a: 26898219.5.0000.5149). Written informed consent was obtained throughout an online formulary that was sent to and signed by all participants right before the phone interview 4099999.

## Results

Out of 118 women who initially agreed to participate, 96 completed the study. Fifteen did not attend the doctor's office and 7 were excluded due to the ineligibility criteria: post-CS abdominal surgery (3), current pregnancy (2), and recent aesthetic scar treatment (2). The participants had a mean age of 34.3 ± 4.2 years. The majority were married (89.6%), of European ethnicity (64.6%), and well-educated with post-graduate qualifications (56%). Most had a healthy BMI (49%), no chronic diseases (90.6%), and were non-smokers (94.8%). The average duration since their last CS was 18 ± 9.4 months, with scar lengths averaging 13.4 ± 1.8 cm. A significant proportion had their first CS (65.6%), and more than half underwent non-elective CS (51%). Regarding aesthetic treatment for CS skin scars, over half of the women (53.1%) expressed occasional interest, while over a quarter (26%) showed a consistent desire for improvement (Table 1).

**Table 1:** Demographic, clinical, and surgical characteristics

Characteristics		p-value
Age (years)		34.3 ± 4.2
Marital status		
	Married	86(89.6)
	Single	8(8.3)
	Divorced	2(2.1)
Ethnicity		
	European (White)	62(64.6)
	Indigenous	27(28.1)
	African ethnicity	7(7.3)
Educational level		
	Completed elementary school	1(1)
	Completed high school	16(16.7)
	Completed an undergraduate qualification	21(21.9)
	Postgraduate/Specialist	54(56)
	Master's or Doctoralate degree	4(4.2)
BMI (kg/m²)		
	Underweight(<18.5)	20(20.8)
	Normal weight(18.5-24.9)	47(49)
	Overweight(25.0-29.9)	29(30.2)
	Obesity(>30.0)	0
Chronic disease		
	Asthma	2(2)
	Autoimmune disease	1(1)
	Hypothyroidism	3(3.1)
	Hypercholesterolemia	1(1)
	Sinusitis	2(2)
	No chronic disease	87(90.6)
Active smoker		
	Yes	5(5.2)
	No	91(94.8)
Are you interested in aesthetic scar treatment?		
	Never	20(20.8)
	Sometimes	51(53.1)
	Always	25(26.0)
Postoperative month (mean±SD)		18.1± 9.4
Scar lenght (cm)		13.4± 1.8
Previous cesarean section		
	0	63(65.6)
	1	31(32.3)
	2	2(2.1)
Timing of surgery		
	Non-elective	49(51)
	Elective	47(49)
Health system		
	Public Health System (SUS)	0(0)
	Private Health System	96(100)

SD, standard deviation; SUS, Sistema Único de Saúde (Brazilian public health system); BMI, body mass index

The PSAQ revealed that "appearance" (mean score = 19.1 ± 3.5) and "satisfaction with appearance" (mean score = 16 ± 3.6) were the main concerns regarding scar characteristics, as "perception" (mean score = 10.8 ± 3.8) and "satisfaction with simptoms" (mean score = 5.6 ± 2.2) weren´t major problems (Table 2).

**Table 2:** Patient scar assessment questionnaire sub-scales

Sub-scales	Best possible score	Worst possible score	Mean study score (mean ± SD)
Appearance	9	36	19.1 ± 3.5
Perception	6	24	10.8 ± 3.8
Satisfaction with appearance	8	32	16 ± 3.6
Satisfaction with symptoms	5	20	5.6 ± 2.2
Total score	28	112	51.5

The POSAS revealed an average score of 26.77 ± 7.49 on the OSAS and 22.9 ± 8.73 on the PSAS. The PSAS identified ‘irregularity’ and ‘thickness’ as the most concerning scar characteristics. Similarly, ‘irregularity’ and ‘pigmentation’ were the most noted issues on the OSAS. These findings suggest a consensus on the primary scar concerns from both the patient and the observer perspectives (Table 3).

**Table 3:** Patient and Observer scar assessment scale

Items	Observer (mean score ± SD)	Patient (mean score ± SD)
Vascularity	3.17	NA
Pigmentation	4.86	4.86
Thickness	4.43	5.28
Irregularity	5.32	5.34
Pliability	4.54	4.32
Surface area	4.42	NA
Itching	NA	1.62
Pain	NA	1.55
Total score	26.77 (±7.49)	22.9 (±8.73)

NA, not applicable. SD, standard deviation

**Figure 1:**
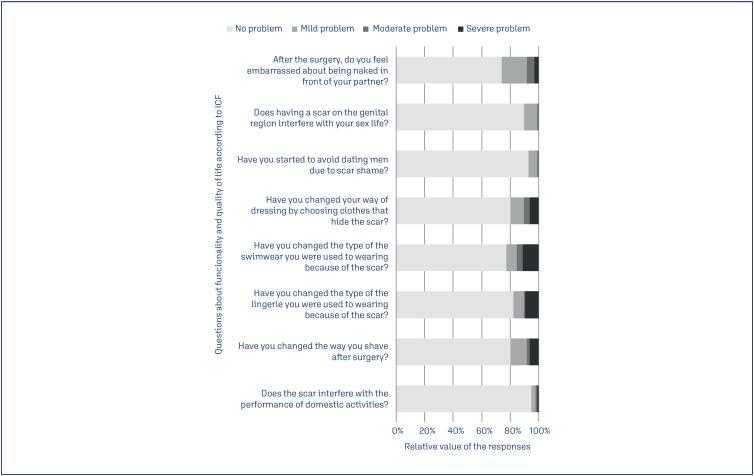
Results of the WHO's International Classification of Functionality, Disability, and Health (ICF) derived questionnaire

The ICF-derived questionnaire indicated that "severe problems" were most frequently reported in the domain of "self-care". Specifically, changes in swimwear and lingerie due to CS scars were noted by 11.4% and 9.4% of participants, respectively. In the domain "interpersonal interactions and relationships", 10.4% of women reported a severe or a moderate impact of the scar on being naked in the front of their partner. In contrast, 89.5% and 92.7% of participants reported no problems on their sex life and on dating, respectively. The domain "domestic life" was the least affected, with 94.8% reporting no problems on the performance of domestic activities (Figure 1).

**Figure 2:**
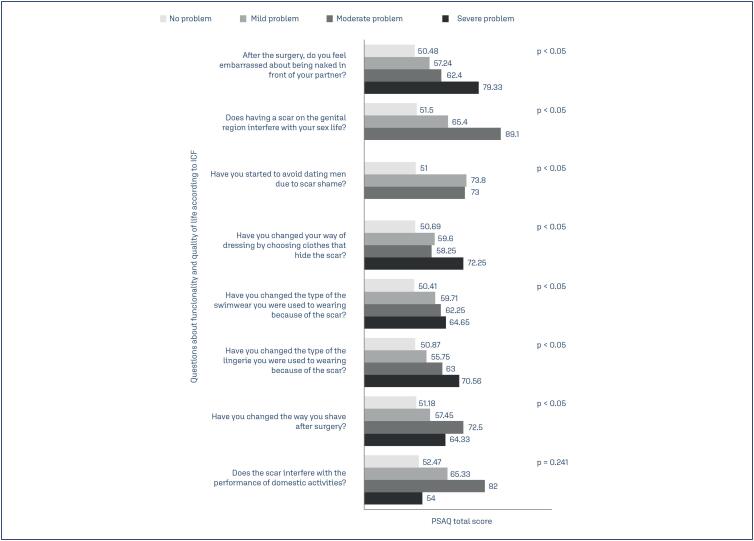
Association beetween funcionality according to WHO's International Classification of Functionality, Disability, and Health (ICF) derived questionnaire and the total Patient Scar Assessment Questionnaire (PSAQ) score. The total PSAQ score ranged from 28 (best score) to 112 (worst score)

**Figure 3:**
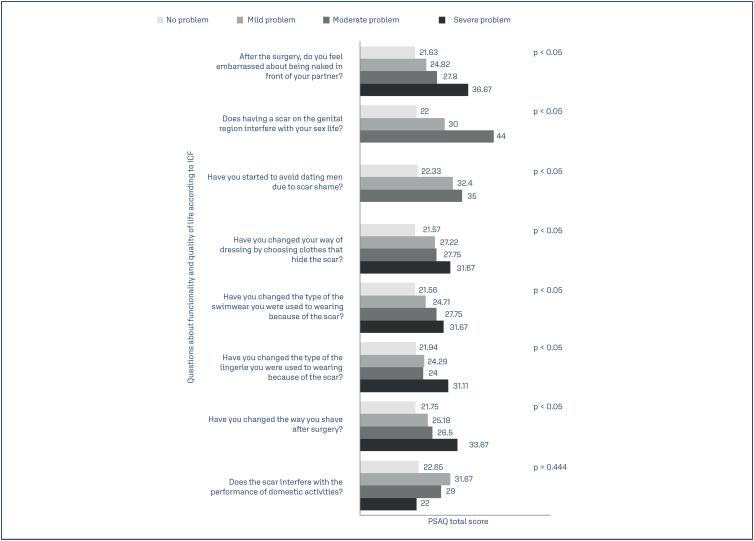
Association beetween funcionality acording to WHO's International Classification of Functionality, Disability, and Health (ICF) and total Patient Scar Assessment Scale (PSAS) score. The total PSAS score ranged from 6 (best score) to 60 (worst score)

There was a statistically significant correlation between the total score of the PSAQ and of the PSAS and the functional impact on ‘interpersonal interactions and relationships’ and ‘self-care’ activities (p < 0.05). The results suggest that the CS scars that possess the worst possible physical attributes on the PSAQ an on the PSAS are the most likely to negatively influence multiple daily activities such as being naked in front of your partner, having a sex life, dating, choosing clothes, swimwear and lingeries and shaving. No statistically significant correlation was found beetween the "domestic life" domain and the total score of the PSAQ (p = 0.241) and of the PSAS (p = 0.444) (Figures 2 and 3).

## Discussion

Our research marks the first dedicated exploration into the long-term clinical and functional ramifications of cesarean section (CS) scars, raising questions about its long-term psychosocial effects. Predominantly, dissatisfaction stemmed from the aesthetic displeasure associated with the scar's appearance. This dissatisfaction occasionally extended into the personal aspects of the participants’ lives, compelling some women to alter their choice of swimwear, a decision that reflects the impact of the CS scars in the intimate spheres of life.

While the majority of literature primarily addresses direct medical complications of CS such as puerperal hemorrhages, infections and isthmocele, there is a tendency to overlook the psychological aspects of femininity that are intimately connected with CS outcomes.^(2-4)^ In this matter, it is crucial to acknowledge the substantial correlations this research has established between CS scars and aspects of women's lives that are often marginalized and deserve greater consideration in clinical decision-making, such as self-image and partner perception. These psychosocial dimensions, though frequently discounted in everyday healthcare practices, should be integrated to the dialogue between healthcare providers and expectant mothers or couples when determining the method of delivery. This is particularly pertinent in the context of elective cesarean delivery or ‘cesarean on request,’ a prevalent practice that deeply influences a woman's postpartum well-being.^(14)^

Contrasting our findings with the available literature, the PSAQ has been previously utilized to evaluate the impact of various abdominal scars, indicating that laparoscopic cholecystectomy scars are generally more accepted than CS scars.^(15)^ Similarly, studies comparing abdominal skin scars from conventional colectomy have found comparable levels of dissatisfaction, implying that even less visible scars can still elicit discontent.^(16)^ Our results reflect these sentiments, with POSAS scores aligning closely with those in the literature, thereby underscoring an aesthetic concern that is present across different types of surgical scars.^(16-19)^

Our findings also reveal that CS scars are not mere superficial concerns but catalysts for significant discontent and functional challenges, markedly degrading the life quality of affected women. In a deep analysis concerning the gender differences in wound healing process, although multiple researchers have explored gender variances in skin properties, we identified a glaring scarcity of data on the intersection of gender and postoperative scars. In a more general context, distinct sex- and gender-specific risks in the emergence and management of health conditions have been identified, highlighting critical distinctions that require a nuanced approach to health care. The nuances of these sex and gender differences harbor profound consequences for the orientation of health services and the personalization of medical treatments. With women's health issues still featuring prominently on the incomplete agenda of global health priorities, the perpetuation of unaddressed needs, such as the gender differences in postoperative scars, is evident. Therefore, a systematic integration of sex and gender considerations into the fabric of health policy, educational programs, research initiatives, and data analytics is not only overdue but imperative for bridging these gaps.^(20-22)^

The strength of our study lies in its use of validated, internationally recognized questionnaires that deliver a dual perspective: the patient's subjective experience and the objective observer's assessment. Nonetheless, the study is not without limitations. The sampling, that is mainly white, married, with high educational level, worried about their weight and aesthetics and drawn exclusively from the private healthcare sector, may not reflect the experiences of all the Brazilian population. Additionally, the absence of a comparison group, such as women with episiotomy scars from vaginal deliveries, restricts our understanding of the CS scar's unique impact on delivery method choice.^(12,13,23)^

The implications of our findings are significant for clinical practice and policy formulation. They highlight the necessity for a comprehensive, multidisciplinary approach to post-CS care that addresses both the physical and psychosocial aspects of scarring. With the prevalence of CS, these insights are invaluable for guiding clinical decisions and informing healthcare providers about the potential for postoperative psychosocial distress.^(3,24,25)^

Future research should endeavor to conduct longitudinal studies to track the functional and aesthetic outcomes over time. Moreover, expanding the sample to include public healthcare recipients could provide a more representative understanding of CS scarring's impact.

## Conclusion

Our study reveals that CS scars, while clinically manageable, can exert a profound influence on women's functionality and self-image, necessitating a holistic approach to postoperative care. Going forward, it is imperative that healthcare providers consider these findings in their practice, and that future research continues to seek improved scar management strategies to mitigate the identified effects.
